# The Dual Role of the Medicinal Mushroom *Fomitopsis pinicola* in Inhibiting Biofilm and Reducing Antibiotic Resistance of Methicillin‐Resistant *Staphylococcus aureus*

**DOI:** 10.1002/fsn3.70355

**Published:** 2025-05-27

**Authors:** Başar Karaca, Noah Kyalo Kilonzo, Şilan Korkmaz, Okan Onar, Özlem Yıldırım, Arzu Çöleri Cihan

**Affiliations:** ^1^ Department of Biology, Faculty of Science Ankara University Ankara Turkey

**Keywords:** antibiofilm, antimicrobial, cytotoxicity, efflux pump inhibition, *Fomitopsis pinicola*, methicillin‐resistant *Staphylococcus aureus*

## Abstract

This study investigates the antimicrobial, antibiofilm, and anti‐quorum sensing activity of *Fomitopsis pinicola* against methicillin‐resistant *Staphylococcus aureus* (MRSA) strains and its potential to improve the efficacy of conventional antibiotics and exert selective cytotoxic effects on cancer cells. Ethanolic extracts of *F. pinicola* were analyzed for antibacterial activity by MIC and time‐kill assays. Synergistic interactions with antibiotics were quantified using checkerboard assays. Antibiofilm activity was analyzed on polystyrene and stainless‐steel surfaces. Anti‐quorum sensing activity was determined by the inhibition of violacein in *Chromobacterium violaceum* . Efflux pump inhibition was assessed by the accumulation of the ethidium bromide. The down‐regulation of virulence genes (*agrA*, *hla*) was measured by qRT‐PCR (quantitative real‐time reverse‐transcription PCR). FT‐IR (Fourier transform infrared spectroscopy) spectroscopy characterized the bioactive compounds, and the cytotoxicity assays on HT‐29 colon cancer and Vero cells evaluated selective toxicity. The extract showed strong antibacterial effects with a MIC of 312.5 μg/mL and concentration‐dependent bactericidal activity. Synergistic interactions with antibiotics led to FIC indices ≤ 0.5. The extract significantly inhibited biofilm formation and eradicated already formed biofilms. Sub‐MIC concentrations reduced quorum sensing by 85.01%, inhibited efflux pump activity, and down‐regulated virulence‐associated genes. FT‐IR analysis confirmed the presence of triterpenoids and terpenoids. The extract displayed selective cytotoxicity on HT‐29 cancer cells, showing strong inhibition, while normal Vero cells were spared. These results emphasize the potential of *F. pinicola* as a robust candidate for antimicrobial therapeutics, especially against biofilm‐associated and multidrug‐resistant pathogens, as well as a selective anticancer agent.

## Graphical abstract

The ethanolic extract of Fomitopsis pinicola showed potent antibacterial, antibiofilm, anti‐quorum sensing, efflux pump inhibitory, and antivirulence activities against Methicillin‐resistant *Staphylococcus aureus* , significantly downregulating agrA and hla gene expression. Synergistic interactions with conventional antibiotics (FIC ≤ 0.5) and concentration‐dependent bactericidal effects were observed. FT‐IR analysis identified triterpenoids and terpenoids as major constituents. Moreover, the extract showed selective cytotoxicity by significantly inhibiting HT‐29 colon cancer cells while exhibiting minimal toxicity towards normal Vero cells, emphasizing its therapeutic potential.

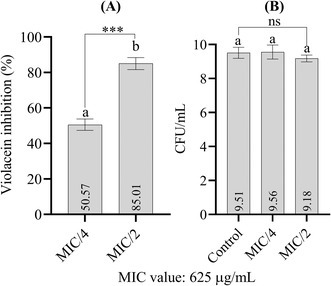

## 1 Introduction

*Fomitopsis pinicola* is a well‐known medicinal mushroom that has been used for many years in traditional Chinese medicine and Korean folk medicine. This mushroom, which is believed to be non‐toxic, also has many clinical effects. In recent years, interest in the medicinal use of *F. pinicola* has increased. The fruiting body of *F. pinicola* contains many different chemical components. Various studies have shown that the components isolated from the fruiting bodies have different pharmacological effects (Zahid et al. 2020). The active components of *F. pinicola* have antitumor, antidiabetic, antioxidant, antimicrobial, and anti‐inflammatory effects (Keller et al. 1996; Choi et al. 2007; Cha et al. 2009).

The steroids, lanostane triterpenoids, and ergostane contained in *F. pinicola* have strong antimicrobial properties (Keller et al. 1996; Liu et al. 2010). Ethanol, ethyl acetate, and methanol extracts of *F. pinicola* show inhibitory effects on *S. aureus* strains as well as on Gram‐negative species such as *Escherichia coli* , *Klebsiella pneumoniae* , and *Pseudomonas aeruginosa* (Dresch et al. 2015; Pala et al. 2019). Huguet et al. (2022) reported that methanol and ethyl acetate extracts of *F. pinicola* had remarkable antimicrobial effects against multidrug‐resistant *E. coli* and *S. aureus* strains isolated from clinical settings.

Natural products are increasingly being investigated for their potential to combat microbial resistance and biofilm‐associated infections. The urgency to develop alternative strategies against multidrug‐resistant (MDR) pathogens such as methicillin‐resistant *Staphylococcus aureus* (MRSA) has fueled interest in natural extracts with broad‐spectrum antimicrobial activity (Aldarhami et al. 2023). Only a few limited studies on the antibiofilm potential of *Fomitopsis* have shown promising antibiofilm properties against microbial pathogens. The antibiofilm activity of this fungus is attributed to its abundant supply of bioactive metabolites, including terpenoids, polysaccharides, and phenolic compounds. These compounds can interfere with quorum sensing mechanisms and disrupt the formation of extracellular polymeric substances (EPS), both of which are crucial for biofilm stability and microbial adhesion (Prajapati et al. 2023; Vunduk et al. 2023; Roychoudhury et al. 2024).

Recent studies have shown that the combination of medicinal mushroom extracts with conventional antibiotics is promising when it comes to improving antimicrobial efficacy and overcoming the increasing challenge of antibiotic resistance. It has been shown that bioactive compounds from medicinal mushrooms can enhance the influence of antibiotics against resistant bacterial strains. These compounds could also have synergistic effects by increasing antibiotic uptake and disrupting bacterial resistance mechanisms, thus improving treatment outcomes against multi‐drug resistant pathogens (Tinrat 2015; Hong et al. 2016; Chatterjee et al. 2016).

MRSA is a common etiologic factor not only in nosocomial infections in the clinical setting but also in community‐acquired infections and may be associated with conditions such as bacteremia, endocarditis, and skin or soft tissue infections (Turner et al. 2019). MRSA is primarily resistant to β‐lactam antibiotics but can also be resistant to many other antimicrobial agents (Bakthavatchalam et al. 2019). The ability of MRSA strains to form biofilms is also a key factor that contributes to their virulence (Kawamura et al. 2011). For example, MRSA biofilms are the main risk factor for skin and soft tissue infections (King et al. 2006). Therefore, targeting MRSA biofilms with natural medicinal agents could pave the way for new therapeutic developments against MRSA infections.

In this study, *S. aureus* ATCC 25923 and ATCC 43300 (MRSA) were selected as model organisms to investigate the antimicrobial, antibiofilm, and antivirulence effects of the *F. pinicola* extract. These strains were selected due to their clinical relevance, their well‐characterized ability to form biofilms, and their contrasting antibiotic susceptibility profiles, which provide comparative insights into methicillin‐susceptible and resistant phenotypes. MRSA in particular poses a serious therapeutic challenge due to its multidrug resistance (MDR) and biofilm‐associated persistence. While *F. pinicola* has a broad spectrum of antimicrobial activity, this study focuses on Gram‐positive *S. aureus* strains to investigate the targeted efficacy of the extract against biofilm‐associated infections commonly found in hospital and community settings.

Given the urgent need for new therapeutic strategies against MRSA, this study aims to investigate the antibiofilm and antimicrobial effects of *F. pinicola* extract on *S. aureus* strains. It was hypothesized that the bioactive compounds containing *F. pinicola* may inhibit the formation of MRSA biofilms and improve the efficacy of conventional antibiotics against these pathogens. This study aims to provide insights into potential alternative treatments that could alleviate the impact of MRSA infections and address the growing challenge of antibiotic resistance by evaluating the synergistic effects of *F. pinicola* extract in combination with antibiotics.

## 2 Materials and Methods

### 2.1 The Mushroom Sample and Its Extraction Procedure

The specimen of *F. pinicola* was deposited in the herbarium of Ankara University. After identification and labeling, the specimen was carefully cleaned and dried to prepare it for the extraction process.

For the extraction process, the specimens were ground to powder with liquid nitrogen. From the resulting powder, ethanolic extracts were prepared according to the protocol described by Onar et al. (Onar et al. 2016). The freeze‐dried powder was dissolved in 70% ethanol to reach a final concentration of 10 mg/mL and stored at −20°C for subsequent analyses.

### 2.2 Bacterial Strains

Methicillin‐resistant *S. aureus* ATCC 43300 (MRSA) and *S. aureus* ATCC 25923 strains were used in all experimental studies. The strains were cryo‐protected with 60% glycerol containing TSB at −86°C and revived by inoculation onto Tryptic Soy Agar (TSA; Merck, Germany) plates, followed by overnight incubation at 37°C before each experiment.

### 2.3 Antimicrobial Activity Tests

#### 2.3.1 Agar Well Diffusion Test

The culture suspensions for the test strains were prepared according to the guidelines of the Clinical and Laboratory Standards Institute (CLSI 2006). In brief, individual colonies of each test strain grown on TSA plates were harvested and inoculated onto Mueller‐Hinton agar (MHA; Merck, Darmstadt, Germany) plates. After incubation at 37°C during 18–24 h, single colonies were collected and suspended in 0.9% NaCl to achieve a turbidity equivalent to the 0.5 McFarland standard.

The prepared culture suspensions were spread evenly onto MHA plates using sterile cotton swabs. 6 mm wells were prepared aseptically, and 100 μL of the extract solution at a concentration of 5 mg/mL was added to the wells. The plates were incubated at 37°C for 18–24 h. The diameters of the inhibition zones were then recorded in mm.

#### 2.3.2 Minimum Inhibitory Concentration Test

The culture suspensions were prepared as previously described. The broth microdilution method was performed with slight modifications based on the CLSI guidelines (CLSI 2006). Extract concentrations from 5000 to 9.77 μg/mL were prepared in sterile Mueller‐Hinton broth (MHB; Merck, Darmstadt, Germany) using a two‐fold serial dilution technique. Wells containing only the medium served as negative controls, while wells consisting of both the medium and the inoculum served as positive controls. After incubation at 37°C for 18–24 h, the lowest concentration of extract that inhibited microbial growth was recorded as the minimum inhibitory concentration (MIC) value.

#### 2.3.3 Time‐Kill Assay

This test was performed with slight modifications as described by Leber (2020). Prior to the test, active cultures of the test strains were prepared overnight in Mueller‐Hinton broth (MHB). The colony‐forming units per milliliter (CFU/mL) of these cultures were calculated beforehand, resulting in approximately 10^9^ CFU/mL for each strain. After incubation, cultures were inoculated into tubes containing 5 mL of fresh MHB supplemented with extract concentrations of 4 × MIC, 2 × MIC, and MIC. The final population density of each suspension was approximately 10^8^ CFU/mL. Tubes containing only media and inoculum were designated as control groups.

The culture suspensions were incubated at 37°C, and serial dilutions were performed by sampling 100 μL of each culture at specific time points (0, 5, 10, 15 and 30 min; 1, 2, 4 and 24 h). Colonies were counted by plating on TSA plates, which were then incubated at 37°C for 24 h.

The colony counts of the control group at time zero (*T*_0_) were compared with those of the test groups at different time points. The percentages of e logarithmic reduction were determined using the formula in the following equation (1), where LR denotes the logarithmic reduction. Finally, a reduction of ≥ 99.9% (≥ 3 log_10_ CFU/mL) compared to the control group was considered the minimum threshold for bactericidal activity.

(1) $$\left[\left(1-10^{-\mathrm{LR}}\right)\right]\times 100$$

### 2.4 *In vitro* Evaluation of *F. pinicola* Extract With the Combination of Antibiotics

The MICs of chloramphenicol, ampicillin, kanamycin, oxacillin, and streptomycin for MRSA ATCC 43300 were determined using the microdilution method (CLSI 2006). The antimicrobial effect of the *F. pinicola* extract in combination with the antibiotics was evaluated using a checkerboard test. Different concentrations of antibiotics were added to the wells of the microtiter plate containing different concentrations of *F. pinicola* extract in MHB media by serial dilution. In addition, the different concentrations of antibiotics and extract were applied separately into the wells. The inocula were prepared according to CLSI recommendations. The treated plates were then incubated at 37°C for 18–24 h. The values of the Fractional Inhibitory Concentration (FIC) Index were calculated using the following formula in the following equations ((2), (3), (4)).

(2) $$\mathrm{FIC}\;\text{extract}=\left(\mathrm{MIC}\;\text{extract in combination}\;\right)÷\left(\mathrm{MIC}\;\text{extract alone}\right)$$

(3) $$\begin{array}{c} \mathrm{FIC}\;\text{antibiotic}=\left(\mathrm{MIC}\;\text{antibiotic in combination}\right)\\ ÷\left(\;\mathrm{MIC}\;\text{antibiotic alone}\right) \end{array}$$

(4) ΣFIC=FICextract+FICantibiotic

The combined effect of the extract and antibiotics was classified as “synergistic” when their total FIC (ΣFIC) did not exceed 0.5, as “indifferent” at ΣFIC values between 0.5 and 4.0, and as “antagonistic” when the ΣFIC reached 4.0 or more.

### 2.5 Antibiofilm Activity Tests

#### 2.5.1 Biofilm Formation on Polystyrene Surfaces

The antibiofilm activity of the *F. pinicola* extract was evaluated using two different approaches. In the co‐treatment approach, the effect of the extract at MIC and sub‐MIC concentrations (MIC/2, MIC/4, … MIC/64) on biofilm formation was investigated. To stimulate biofilm formation by *S. aureus* ATCC 43300 and *S. aureus* ATCC 25923, the previously established conditions were followed as described by Onbas et al. (2019). Specifically, biofilm formation was performed in Tryptic Soy Broth (TSB; Merck, Darmstadt, Germany) supplemented with 3% NaCl and 0.5% glucose.

A total of 135 μL of the modified medium was added to each well of a 96‐well microtiter plate. The test wells were spiked with the extract to obtain MIC and sub‐MIC concentrations for each strain. Wells containing only the medium and wells containing both the medium and the inoculum were designated as negative and positive controls, respectively. To prepare the inoculum, the cultures that had grown overnight at 37°C in the modified medium were diluted 1:10 to achieve a concentration of approximately 10^8^ CFU/mL. Subsequently, 15 μL of the diluted culture suspension was added to each well of the positive control and test groups. The plates were incubated for 24 h at 37°C. After incubation, the crystal violet binding assay was performed to evaluate biofilm formation (Onbas et al. 2019). Optical density measurements were recorded at 595_nm_ using an ELISA reader (BioTek Elisa Reader, μQuant, BioTek Inc., Winooski, VT, USA). The inhibition of biofilm formation was calculated as a percentage inhibition with the formula given in equation (5).

(5) $$\text{Inhibition}\;\left(\%\right)=\left[\left(C-B\right)-\left(T-B\right)\right]÷\left[\left(C-B\right)\right]\times 100$$

(*C*, well including the inocula; *B*, well including the culture media; *T*, well including the inocula, media, and the active substance).

In the post‐treatment, the extract was tested in concentrations of MIC/2, MIC, 2 × MIC, and 4 × MIC on previously established biofilms. First, biofilms were developed in 96‐well microtiter plates by incubating them at 37°C for 24 h. After biofilm formation, the wells were washed three times with sterile PBS (phosphate buffered saline, pH 7.4) to remove non‐adherent cells. Subsequently, 150 μL of the extract suspensions prepared in the modified medium were added to the wells. For the negative and positive controls, only the modified medium was added. The plates were then incubated at 37°C for a further 24 h. After the incubation period, the crystal violet binding test was performed again to assess biofilm formation.

#### 2.5.2 Biofilm Formation on Stainless Steel Surfaces

The antibiofilm activity of the *F. pinicola* extract was also tested on stainless‐steel surfaces. In this context, sterile stainless‐steel discs with a diameter of 14 mm (grade 316 L) were used for biofilm sampling both during co‐treatment and post‐treatment. Before the biofilm tests, the discs were pre‐treated by soaking them overnight in isopropanol and then shaking them for 30 min in a chlorinated cleaning solution (Presept effervescent tablets, Johnson & Johnson, Paranaque City, Philippines). After treatment, the discs were thoroughly rinsed with deionized water and sterilized by autoclaving (Kilic et al. 2017). Two of these sterile surfaces were then aseptically placed in each well of a six‐well microtiter plate containing 5 mL of the modified TSB.

Modified media with the MIC, MIC/2, MIC/4, and MIC/8 concentrations of the extract were used for the pre‐treatment test. Inoculation was performed as previously described, and 500 μL of the culture suspensions were inoculated into each well. The wells without the extract, including the inoculum and the modified media, and the wells containing only the culture media were designated as positive and negative control groups, respectively. The plates were then incubated at 37°C for 24 h. The discs in the wells were inverted at the end of the first 12 h to maximize biofilm formation. After 24 h, the discs were aseptically removed and transferred to test tubes containing 5 mL of physiological saline solution (0.9% NaCl) and sterile glass beads (diameter: 3 mm). The test tubes were vortexed at maximum intensity for 2 min. Finally, the suspensions were diluted and spread on TSA agar plates to perform the spread plate assay. The plates were incubated at 37°C for 24 h for colony enumeration. The results were expressed as colony‐forming units per unit area (CFU/cm^2^) and then converted to logarithmic values (log CFU/cm^2^). The % log reduction values were calculated as previously described.

For the post‐treatment test, biofilm samples were developed on the stainless‐steel discs for 24 h. The biofilms were then treated with extract concentrations of MIC/2, MIC, 2 × MIC, and 4 × MIC prepared in the modified medium. After a further 24‐h incubation, the steps described above were carried out to collect and count the biofilm cells and to calculate the log reduction (equation 1).

### 2.6 In vitro Anti‐Quorum Sensing Activity Test

The anti‐quorum sensing activity of the extract was investigated using *Chromobacterium violaceum* ATCC 12472 as the reporter strain according to an adapted protocol by Batohi et al. (2021). First, the MIC of the extract was determined. Subsequently, the strain was cultured in Luria‐Bertani (LB) broth (Merck, Darmstadt, Germany) and in LB supplemented with sub‐MIC concentrations of the extract. After incubation at 30°C for 24 h, 1 mL aliquots were harvested and centrifuged (10,000 × g, 5 min). The pellets were resuspended in 1 mL dimethyl sulfoxide (DMSO), and violacein production was quantified by measuring absorbance at 585 nm. These absorbance values were then used to calculate the rate of violacein inhibition (equation 6).

(6) $$\begin{array}{c} \text{Violacein inhibition}\;\left(\%\right)=[\left(\mathrm{OD}585\text{nmControl}-\mathrm{OD}585n\text{mTest}\right)\\ ÷\left(\mathrm{OD}585\text{nmControl}\right)]\times 100 \end{array}$$

### 2.7 Efflux Pump Inhibition: Agar Cartwheel Method

The inhibition of the efflux pump was monitored according to the slightly modified method of Martins et al. (2013). Methicillin‐resistant *S. aureus* ATCC 43300 was used, and the MIC concentration of EtBr (ethidium bromide) was determined prior to the experiment (MIC_EtBr_: 5 μg/mL). TSA plates were prepared with sub‐MIC concentrations of the extract (78.13 and 156.25 μg/mL) and EtBr (2.5 μg/mL). The plates without the extract but containing the EtBr were also prepared as negative groups. Overnight culture of the strains was suspended in physiological saline, and the culture suspension was prepared according to the 0.5 McFarland standard. 20 μL of the suspensions were dropped onto the plates, and the plates were then incubated at 37°C for 24 h. After incubation, EtBr fluorescence was visualized using the Vilber Lourmat Quantum ST4 gel documentation system (Vilber Lourmat, Marne‐la‐Vallée, France). The relatively increased fluorescence was considered to be an inhibition of the efflux pump.

### 2.8 Quantification of Virulence Gene Expressions by Real Time‐PCR

The expression of virulence genes was evaluated after the treatment of *S. aureus* strains with sub‐MIC values of the extract (MIC/2: 156.25 μg/mL and MIC/4: 78.13 μg/mL). The control groups without extract and the test groups with sub‐MIC treatments were cultured in TSB medium. After incubation, the cells were collected by centrifugation (9000 rpm, 10 min, 4°C). Total RNA was isolated using PureZOL RNA isolation reagent according to the manufacturer's protocol (Bio‐Rad, Hercules, CA, USA). The RNA samples (200 ng/reaction) were then transcribed into cDNA using the iScript cDNA Synthesis Kit (Bio‐Rad). Quantitative real‐time PCR (qRT‐PCR) was performed on a Bio‐Rad CFX96 Touch Real‐Time PCR System using the iTaq Universal SYBR Green Supermix Kit (Bio‐Rad) and specific primers for the target genes: *agrA* (Forward: 5′‐CTACAAAGTTGCAGCGATGGA‐3′, Reverse: 5′‐TGGGCAATGAGTCTGTGAGA‐3′) *hla* (Forward: 5′‐ACAATTTTAGAGCCCAACTGAT‐3′) (Sully et al. 2014), and 16S *rRNA* (Forward: 5′‐TAACTTCGGGAAACCGGAGC‐3′, Reverse: 5′‐GCATCGTTGCCTTGGTAAGC‐3′, this study). Gene expression values were normalized to the housekeeping gene 16S *rRNA* and expressed as *Ct* values. Fold changes in gene expression were calculated using the 2^−ΔΔ*Ct*^ method.

### 2.9 FT‐IR Spectroscopy

An FT‐IR spectrophotometer (IRAffinity 1; Shimadzu IRTracer, 100, Kyoto, Japan) was used to record the percentage transmittance of the *F. pinicola* extract powder in the range of 600–4000 cm^−1^. The first derived spectra were generated to evaluate the relative absorption intensities, while the second derived spectra were used to identify absorption maxima. Each sample was scanned 100 times at a resolution of 4 cm^−1^ under ambient conditions, and the averaged results were then subjected to data processing. The spectra were then discretized in 2 cm^−1^ steps, resulting in 1868 individual data points. Further refinement of the peak positions was performed using the manufacturer's IR Solutions Control Software (Shimadzu, Kyoto, Japan).

### 2.10 Cell Viability Assay

The HT‐29 (ATCC HTB‐38) colorectal cancer and Vero (ATCC CCL‐81) normal epithelial cell lines used in this study were obtained from ATCC. HT‐29 and Vero cells were cultured in RPMI‐1640 medium supplemented with 10% FBS, 1% L‐Glutamine, and 100 U/mL penicillin/streptomycin. Cells were grown in a humidified incubator at 37°C and 5% CO_2_. Cells were used in 10–20 passages and tested negative for mycoplasma contamination.

The effect of *F. pinicola* extract on the viability of HT‐29 and Vero cells was analyzed using the MTT assay as previously described (Van Meerloo et al. 2011). In brief, 1 × 10^4^ cells/well were seeded in 96‐well tissue culture plates and incubated for 18 h. After incubation, cells were treated with the ethanolic extract of *F. pinicola* at different concentrations (31.25–1000 μg/mL) and with the vehicle controls for 24 and 48 h. The cytotoxic activity of the *F. pinicola* extract was expressed as an IC_50_ value and calculated using the dose–response inhibition curve. The selectivity index (SI) of the FP extract was then calculated (Indrayanto et al. 2021). All experiments were performed in triplicate using three technical replicates.

### 2.11 Statistical Analysis

Data were expressed as mean ± SD. Statistical differences were assessed by one‐way ANOVA and Tukey's test for post hoc comparisons using GraphPad Prism software (version 8.0, Boston, MA, USA).

## 3 Results

### 3.1 Antimicrobial Activity Tests

#### 3.1.1 Agar Well Diffusion and MIC Tests

**FIGURE 1:**
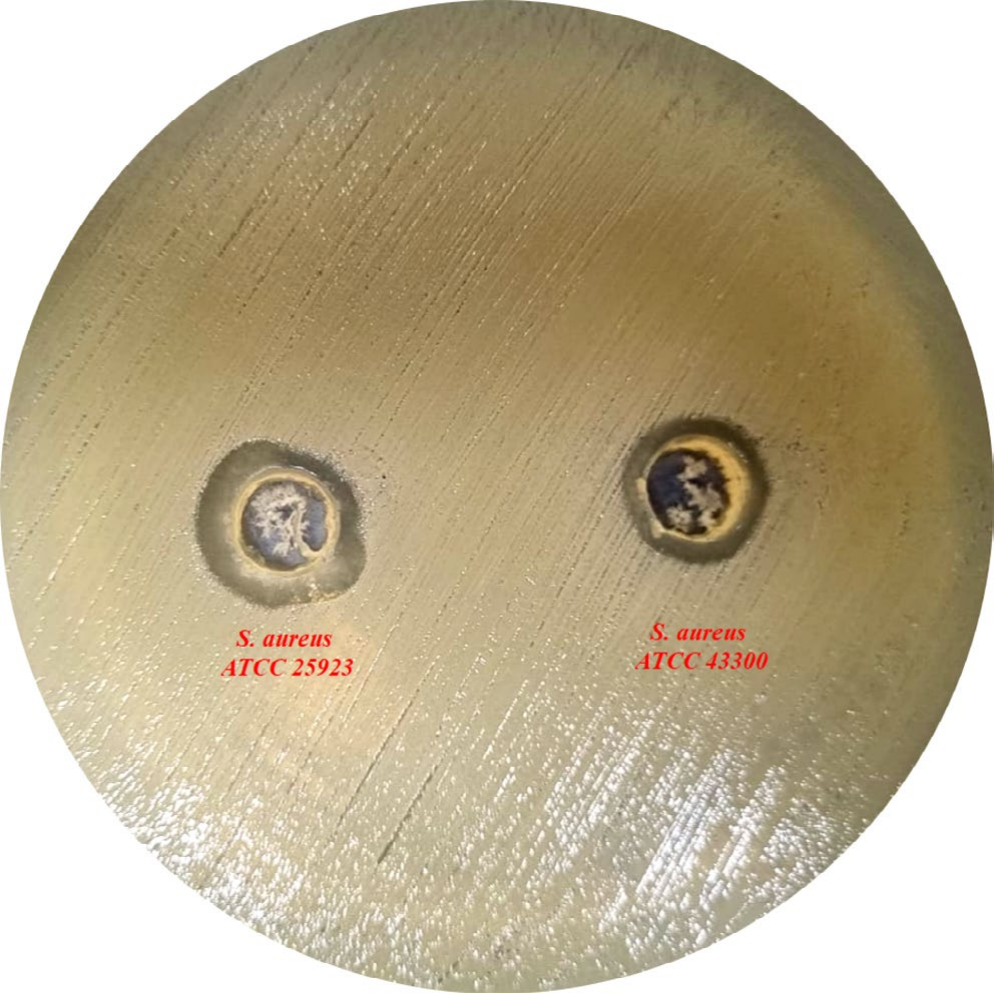
Agar well diffusion test results for the ethanolic extract.

The inhibition zones depicted in Figure 1 show that the ethanolic extract of *F. pinicola* exhibits significant antibacterial activity against *S. aureus* ATCC 25923 and ATCC 43300 strains. The extract was administered at a concentration of 5 mg/mL, while the ethanolic solvent (35%) alone showed no antibacterial activity (data not shown). The MIC of the extract for both strains was determined to be 312.5 μg/mL.

#### 3.1.2 Time‐Kill Assay

**FIGURE 2:**
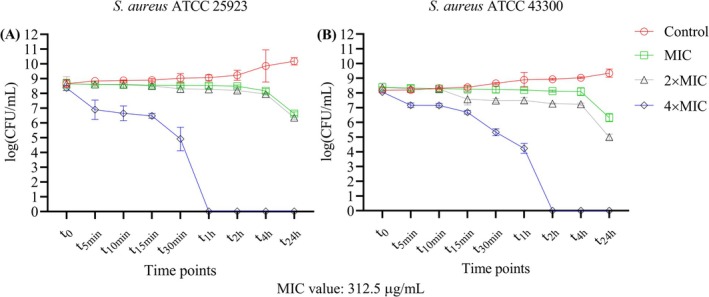
(A) Time‐kill assay results for *S. aureus* ATCC 25923. (B) Time‐kill assay results for *S. aureus* ATCC 43300. The mean values are given with standard deviations. The MIC value is 312.5 μg/mL for both strains.

The time‐kill activity of the ethanolic extract of *F. pinicola* was tested against *S. aureus* strains ATCC 25923 (Figure 2A) and ATCC 43300 (Figure 2B) at concentrations of MIC (312.5 μg/mL), 2 × MIC, and 4 × MIC. Samples were collected at *t*_0_, *t*_5min_, *t*_10min_, *t*_15min_, *t*_30min_, *t*_1h_, *t*_2h_, *t*_4h_, and *t*_24h_ to monitor bacterial survival.

In the control group (untreated group) of *S. aureus* ATCC 25923, bacterial growth followed the typical exponential growth. At the MIC and 2 × MIC concentrations, growth was reduced but not completely inhibited, with a gradual decrease in bacterial survival. In contrast, treatment with the 4 × MIC concentration resulted in a significant reduction in bacterial numbers and showed a rapid bactericidal effect, with viable bacteria being eliminated within 1 h (Figure 2A). With this strain, the first detectable bactericidal effect was achieved with a 99.99% reduction after 30 min at a concentration of 4 × MIC (Table 1).

**TABLE 1:** Percentage log reduction for different MIC values. The MIC value for both strains is 312.5 μg/mL. The mean values are given with standard deviations.

	*S. aureus* ATCC 25923			*S. aureus* ATCC 43300		
	MIC	2 × MIC	4 × MIC	MIC	2 × MIC	4 × MIC
Time points						
*t*_0_	4.43 ± 3.00	1.20 ± 1.18	49.39 ± 5.30	0 ± 0.00	0 ± 0.00	25.53 ± 10.49
*t*_5min_	41.52 ± 2.83	41.97 ± 2.86	98.86 ± 2.99	0 ± 0.00	0 ± 0.00	90.75 ± 1.66
*t*_10min_	46.76 ± 10.35	49.21 ± 3.57	99.41 ± 0.37	5.70 ± 5.11	13.40 ± 2.59	93.25 ± 1.01
*t*_15min_	55.10 ± 2.57	58.74 ± 17.31	99.62 ± 0.18	24.61 ± 15.00	84.72 ± 14.16	98.00 ± 0.01
*t*_30min_	56.72 ± 11.02	74.06 ± 3.18	99.99 ± 0.01	61.72 ± 8.75	93.12 ± 2.37	99.95 ± 0.02
*t*_1h_	71.14 ± 8.49	83.75 ± 3.17	100 ± 0.00	79.68 ± 26.39	96.03 ± 3.72	99.998 ± 0.01
*t*_2h_	81.89 ± 7.81	90.72 ± 3.31	100 ± 0.00	84.42 ± 3.37	97.80 ± 0.27	100 ± 0.00
*t*_4h_	97.96 ± 6.13	98.74 ± 3.96	100 ± 0.00	88.75 ± 6.17	98.42 ± 0.08	100 ± 0.00
*t*_24h_	99.97 ± 0.01	99.98 ± 0.01	100 ± 0.00	99.91 ± 0.08	100 ± 0.00	100 ± 0.00

Similar trends were observed with the strain of *S. aureus* ATCC 43300, with the control showing exponential growth. Treatment with MIC and 2 × MIC decreased the growth but did not completely suppress it. However, treatment with 4 × MIC significantly reduced bacterial counts and achieved complete eradication of the bacteria within 2 h (Figure 2B). For this strain, the first minimal bactericidal effect was observed at the end of the 30 min at a concentration of 4 × MIC, with a reduction of 99.95% being achieved (Table 1).

These results show that the ethanolic extract of *F. pinicola* has a strong bactericidal effect and that the bacterial viability of the two tested strains of *S. aureus* decreases with time and concentration.

### 3.2 *In vitro* Evaluation of *F. pinicola* Extract With the Combination of Antibiotics

Table 2 shows the interpretation of the checkerboard results and the calculated values of the FICI (FIC indices) for the combination of *F. pinicola* extract with different antibiotics. For all combinations, the FIC indices obtained by combining the extract with the tested antibiotics were ≤ 0.5, indicating synergism. The strongest synergism was observed with the combination of the extract and kanamycin.

**TABLE 2:** MIC and FIC values for the combination of different antibiotics and *F. pinicola* extract.

	MIC				FIC			Result
	MIC_antibiotic_ alone (A) (μg/mL)	MIC_antibiotic_ in combination (B) (μg/mL)	MIC_extract_ alone (C) (μg/mL)	MIC_extract_ in combination (D) (μg/mL)	Antibiotic B/A	Extract D/C	Index (B/A) + (C/D)	
Chloramphenicol	8	1	312.50	39.06	0.125	0.125	0.250	Synergism
Ampicillin	256	2	312.50	78.13	0.008	0.250	0.258	Synergism
Kanamycin	256	4	312.50	39.06	0.016	0.125	0.141	Synergism
Oxacillin	256	4	312.50	78.13	0.016	0.250	0.266	Synergism
Streptomycin	16	1	312.50	78.13	0.250	0.063	0.313	Synergism

### 3.3 Antibiofilm Activity Tests

#### 3.3.1 Biofilm Formation on Polystyrene Surfaces

**FIGURE 3:**
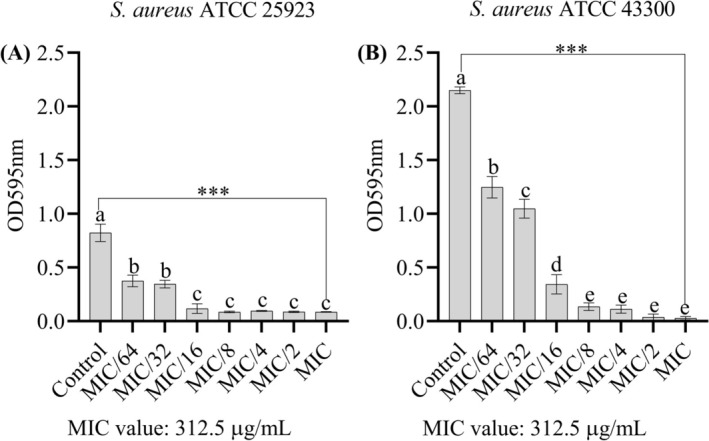
(A) Biofilm formation on polystyrene surfaces: Effect of the extract on *S. aureus* ATCC 25923. (B) Effect of the extract on the biofilm formation of *S. aureus* ATCC 43300. Data are presented as mean ± standard deviation (SD). Statistical differences were assessed using one‐way ANOVA followed by Tukey's post hoc test. ***p < 0.001. Columns not sharing the same lowercase letter indicate statistically significant differences. The MIC value of the extract was determined as 312.5 μg/mL for both strains.

The antibiofilm activity of the ethanolic extract of *F. pinicola* was tested against *S. aureus* strains ATCC 25923 (Figure 3A) and ATCC 43300 (Figure 3B) for biofilm formation at concentrations ranging from MIC (312.5 μg/mL) to MIC/64. Biofilm formation was quantified using crystal violet optical density measurements, and statistically significant differences between treatments were indicated by different letters in the columns.

For strain ATCC 25923, no statistically significant difference in biofilm inhibition was observed between the concentrations of MIC to MIC/16 or MIC/32 to MIC/64. This indicates that the extract has similar antibiofilm activity within these concentration ranges (Figure 3A).

For strain ATCC 43300, the concentrations from MIC to MIC/8 showed no statistically significant differences in antibiofilm activity, indicating a comparable inhibitory effect in this range. However, at MIC/16 and higher concentrations, a significant antibiofilm effect on biofilm formation was also observed. The percentage reduction values for the biofilm formation of the strains are listed in Table 3.

**TABLE 3:** Percentage reduction for the biofilm formation of the strains. The MIC concentration value is 312.5 μg/mL for both strains. The mean values are given with standard deviations.

	Concentrations						
	MIC/64	MIC/32	MIC/16	MIC/8	MIC/4	MIC/2	MIC
*S. aureus* ATCC 25923	54.59 ± 2.39	58.00 ± 4.16	85.83 ± 2.75	89.63 ± 0.85	88.50 ± 0.57	89.51 ± 0.57	89.69 ± 0.58
*S. aureus* ATCC 43300	41.96 ± 2.70	51.27 ± 2.38	84.01 ± 3.12	93.73 ± 1.62	94.77 ± 1.59	98.32 ± 0.06	98.77 ± 0.11

**FIGURE 4:**
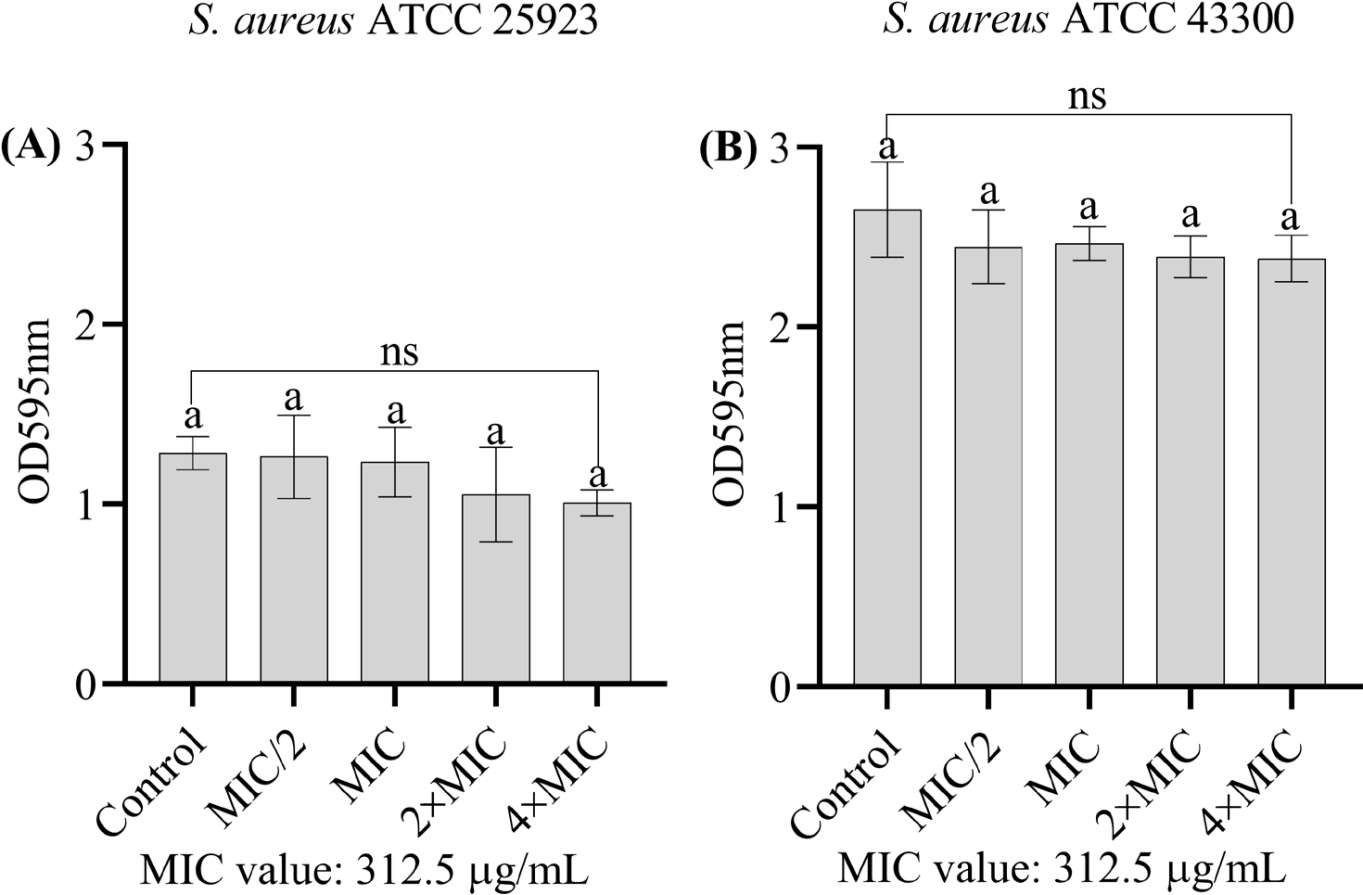
(A) Effect of the extract on pre‐established biofilms of *S. aureus* ATCC 25923 formed on polystyrene surfaces. (B) Effect of the extract on pre‐established biofilms of *S. aureus* ATCC 43300. Data are presented as means ± standard deviation (SD). Statistical differences were determined using one‐way ANOVA followed by Tukey's post hoc test. ns, indicates that there are no statistical differences between the groups. Bars not sharing the same lowercase letter indicate statistically significant differences. The minimum inhibitory concentration (MIC) of the extract was 312.5 μg/mL for both strains.

It was found that the MIC/2, MIC, 2 × MIC and 4 × MIC concentrations of the ethanolic extract of *F. pinicola* were ineffective in removing the biofilm matrix accumulated on polystyrene surfaces in both strains (Figure 4A,B).

#### 3.3.2 Biofilm Formation on Stainless Steel Surfaces

**FIGURE 5:**
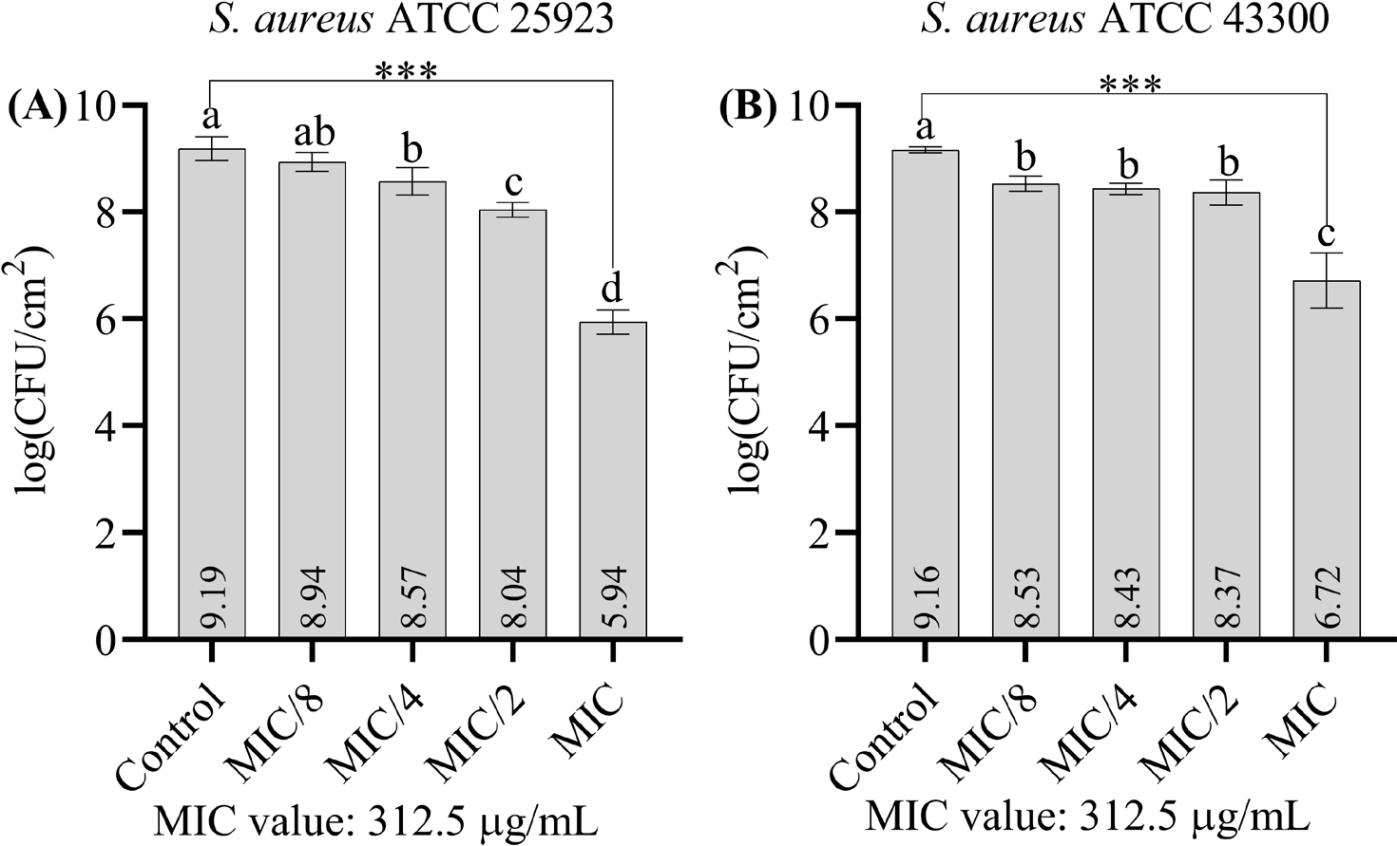
(A) Effect of the extract on the biofilm formation of *S. aureus* ATCC 25923 on stainless steel surfaces. (B) Effect of the extract on the biofilm formation of *S. aureus* ATCC 43300 on stainless steel surfaces. Data are presented as means ± standard deviation (SD). Statistical differences were analyzed using one‐way ANOVA followed by Tukey's post hoc test. ***p < 0.001. Bars not sharing the same lowercase letter indicate statistically significant differences. The MIC value of the extract was determined as 312.5 g/mL for both strains.

In this experiment to evaluate the effects of the ethanolic extract of *F. pinicola* on biofilm formation, the MIC and sub‐MIC concentrations (MIC/2, MIC/4, and MIC/8) were tested. Under the influence of the extract, the biofilm formation of both strains showed a similar trend. While the highest concentration value that inhibited biofilm formation for both strains was determined as the MIC value, significant inhibition was also observed at concentrations below the MIC (Figure 5). The logarithmic reduction values of the biofilm cells are also listed in Table 4.

**TABLE 4:** Effects of the ethanolic extract on biofilm formation. The percentage log reduction values are shown. The MIC concentration is 312.5 μg/mL for both strains. The mean values with standard deviations are shown. The mean values are given with standard deviations.

	Concentrations			
	MIC/8	MIC/4	MIC/2	MIC
*S. aureus* ATCC 25923	43.90 ± 27.56	75.59 ± 12.47	92.84 ± 1.45	99.94 ± 0.05
*S. aureus* ATCC 43300	76.72 ± 4.33	81.24 ± 3.19	83.90 ± 4.11	99.64 ± 0.27

**FIGURE 6:**
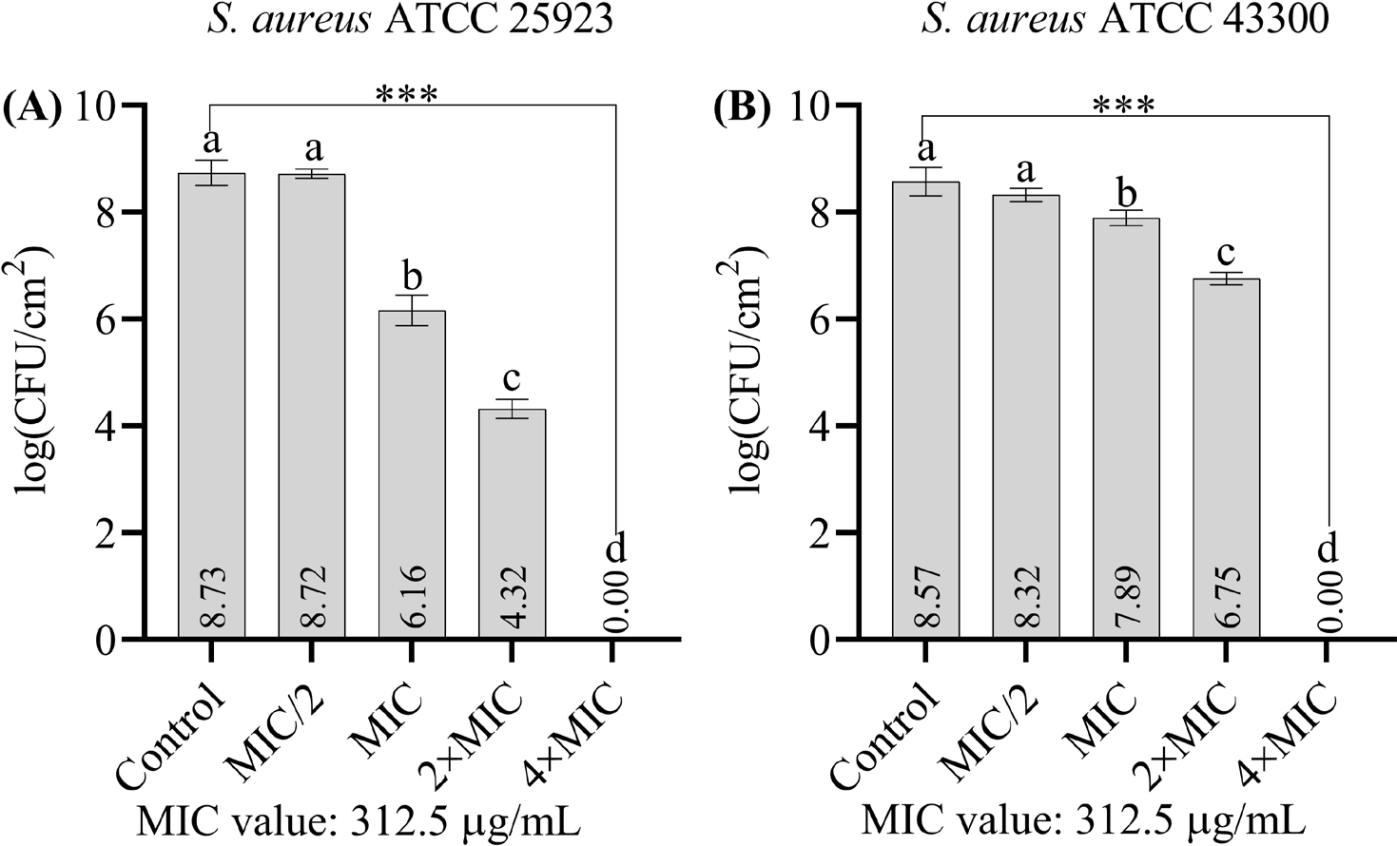
(A) Effect of the extract on pre‐established biofilms of *S. aureus* ATCC 25923 formed on stainless steel surfaces. (B) Effect of the extract on pre‐established biofilms of *S. aureus* ATCC 43300 formed on stainless steel surfaces. Data are presented as means ± standard deviation (SD). Statistical differences were assessed using one‐way ANOVA followed by Tukey's post hoc test. ***p < 0.001. Bars not sharing the same lowercase letter indicate statistically significant differences. The MIC value of the extract was determined as 312.5 μg/mL for both strains.

In the experiment, in which the potential of the extract to eradicate pre‐established biofilms was tested, the concentrations MIC/2, MIC, 2 × MIC, and 4 × MIC were considered. The biofilms of *S. aureus* ATCC 25923 on the stainless steel surface were completely eradicated at 2 × MIC and 4 × MIC concentrations, while the biofilm of *S. aureus* ATCC 43300 was only eradicated at 4 × MIC concentration (Figure 6). When the mature biofilm samples were treated with the MIC concentration, 99.73% and 79.22% eradication was observed for strains *S. aureus* ATCC 25923 and *S. aureus* ATCC 43300, respectively (Table 5).

**TABLE 5:** Effects of the ethanolic extract on pre‐established biofilms. The percentage log reduction values are shown. The MIC concentration is 312.5 μg/mL for both strains. The mean values with standard deviations are shown.

	Concentrations			
	MIC/2	MIC	2 × MIC	4 × MIC
*S. aureus* ATCC 25923	3.39 ± 1.84	99.73 ± 0.12	100 ± 0.00	100 ± 0.00
*S. aureus* ATCC 43300	43.44 ± 18.88	79.22 ± 5.01	98.48 ± 0.31	100 ± 0.00

### 3.4 Anti‐Quorum Sensing Activity

**FIGURE 7:**
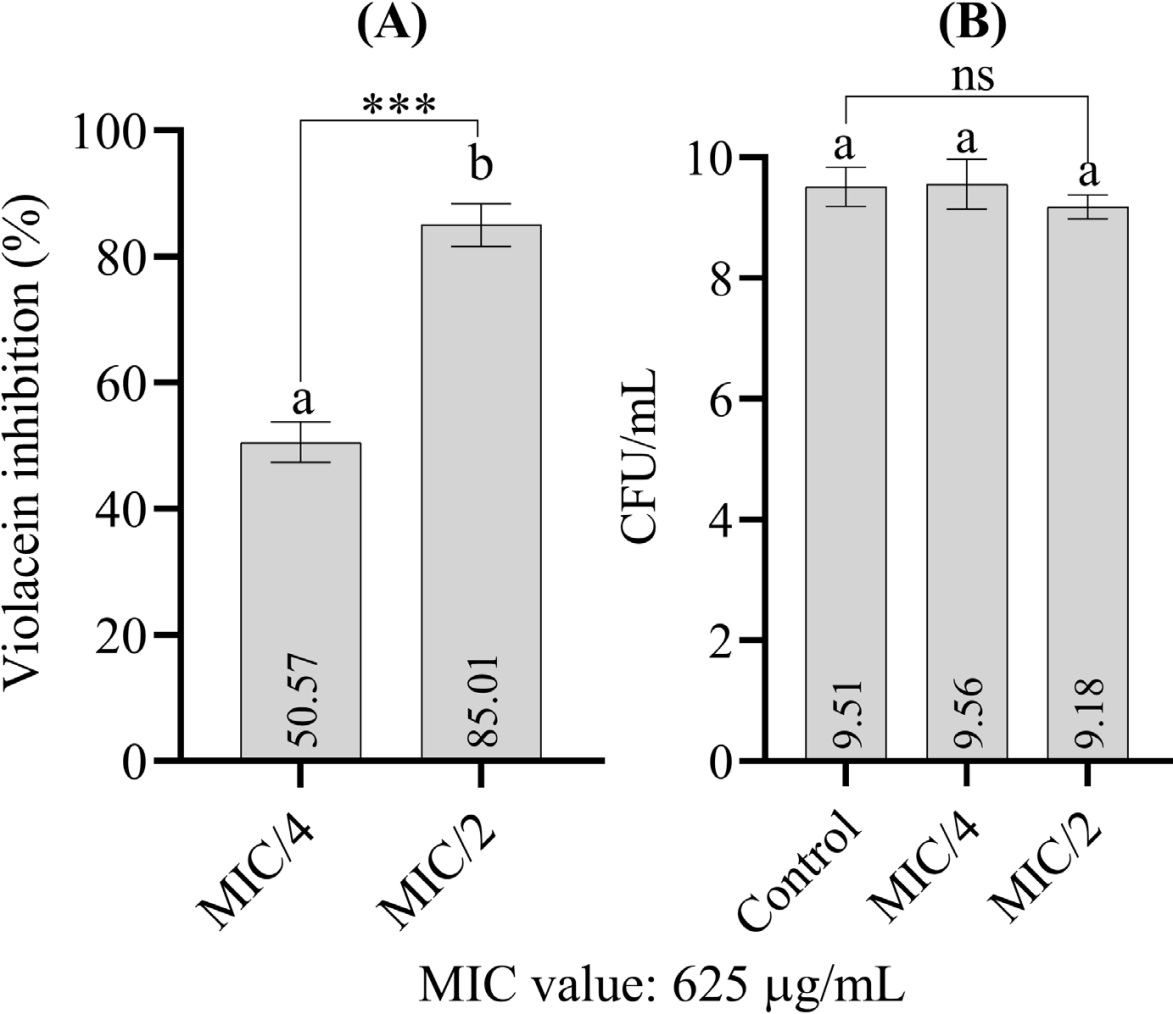
(A) Anti‐quorum sensing activity of the extract, assessed via violacein inhibition in *C. violaceum* . (B) Effect of the tested extract concentrations on bacterial cell viability. Data are presented as means ± standard deviation (SD). Statistical differences for cell viability were evaluated using one‐way ANOVA followed by Tukey's post hoc test. Violacein inhibition was analyzed using a paired sample *t*‐test. ***p < 0.001, ns, indicates that there are no statistical differences between the groups. Bars not sharing the same lowercase letter indicate statistically significant differences.

Figure 7 shows the violacein inhibitory properties of *F. pinicola* at sub‐MIC concentrations (Figure 7A). It was also found that the tested extract concentrations suppressed pigment production without having an inhibitory effect on bacterial cell viability (Figure 7B). The MIC concentration of the extract for *C. violaceum* ATCC 12472 is 625 μg/mL. 312.5 μg/mL (MIC/2) and 156.25 μg/mL (MIC/4) showed a remarkable inhibitory effect on pigment production with percentage reduction rates of 85.01 and 50.57, respectively (Figure 7A).

### 3.5 Efflux Pump Inhibition

**FIGURE 8:**
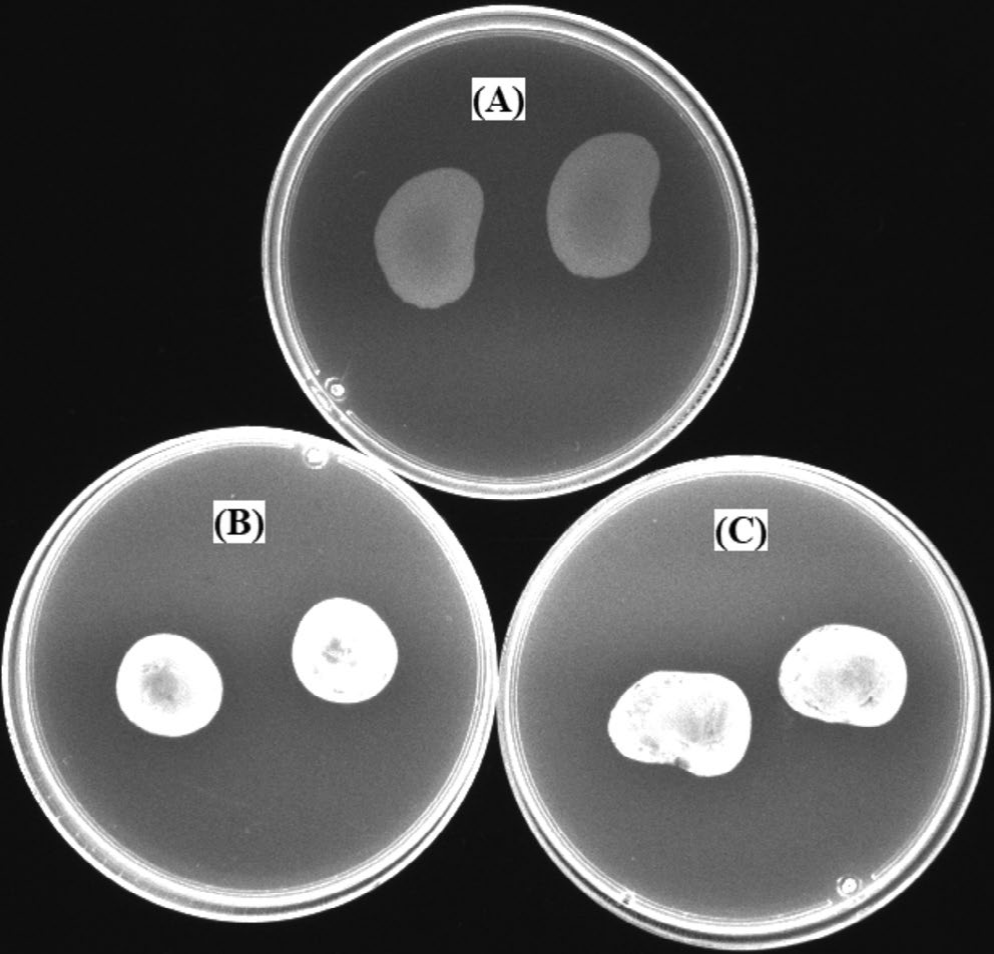
Inhibition of the efflux pump under the effect of *F. pinicola* extract. (A) The control plate with only EtBr (2.5 μg/mL). (B) The test plate with EtBr and 78.13 μg/mL extract (MIC/4). (C) The test plate with EtBr and 156.25 (MIC/2) μg/mL extract.

The increased fluorescence of the EtBr accumulated inside the cells represented the inhibition of the efflux pump (Figure 8). Figure 8A clearly shows that the efflux pump system was active. Since EtBr was an ideal substrate for the efflux pumps, there was weak fluorescence compared to the test plates with the sub‐MIC concentrations of the extract. The increased fluorescence, indicating the disruption of the efflux pump, can be seen in Figure 8B,C.

### 3.6 Quantification of Virulence Gene Expressions by Real Time‐PCR

**FIGURE 9:**
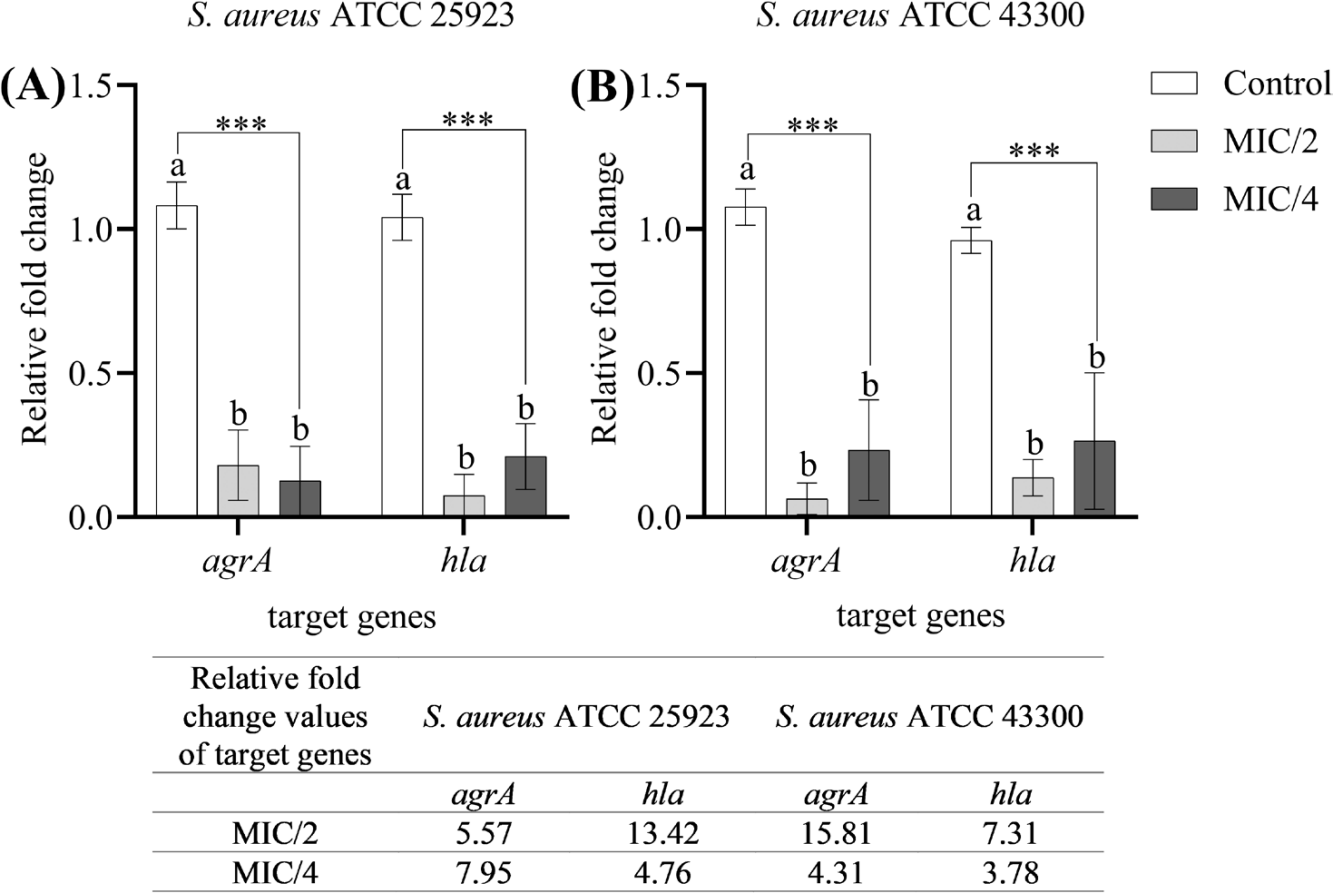
Relative fold change and expression levels of *agrA* and *hla* genes following treatment with sub‐MIC concentrations of the extract. (A) *S. aureus* ATCC 25923. (B) *S. aureus* ATCC 43300. Data are presented as means ± standard deviation (SD). Statistical differences were analyzed using one‐way ANOVA followed by Tukey's post hoc test. ***p < 0.001. Bars not sharing the same lowercase letter indicate statistically significant differences.

Expression levels of virulence‐related genes were assessed by qRT‐PCR, with fold changes calculated using the 2^−ΔΔ*Ct*^ method. Treatment of *S. aureus* strains with sub‐MIC concentrations of *F. pinicola* extract resulted in significant, concentration‐dependent suppression of *agrA* and *hla* expression compared to untreated controls (*p* < 0.001) (Figure 9). It is noteworthy that a significant reduction in gene expression was observed in both MIC/2 and MIC/4. Importantly, this down‐regulation had no effect on bacterial viability as confirmed by CFU counting (data not shown), emphasizing the ability of the extract to inhibit the expression of virulence factors without exerting bactericidal effects.

### 3.7 FT‐IR Spectroscopy

The FTIR spectrum of the ethanolic extract of *F. pinicola* exhibits distinct absorption bands at 3296, 2938, 2352, 1699, 1374, 1242, 1032, and 610 cm^−1^. The broad peak at 3296 cm^−1^ indicates O–H stretching vibrations, suggesting the presence of hydroxyl groups commonly found in alcohols and phenolic compounds. The band at 2938 cm^−1^ corresponds to the C–H stretching vibrations characteristic of aliphatic hydrocarbons. The absorbance at 1700 cm^−1^ is attributed to C=O stretching vibrations, which indicate carbonyl groups typical of ketones, aldehydes, or carboxylic acids as observed in polysaccharides. The peak at 1456 cm^−1^ is attributed to C–H bending in CH_3_ groups. Peaks at 1375 and 1242 cm^−1^ associated with C–H bending and C–O stretching vibrations indicate the presence of polysaccharides or glycosidic bonds. The band at 1033 cm^−1^ corresponds to C–O–C stretching vibrations indicative of ether groups, while the absorption at 611 cm^−1^ could be due to skeletal vibrations typical of terpenoid compounds (see also, Figure S1 for the spectra plot).

### 3.8 Cell Viability Assay

**FIGURE 10:**
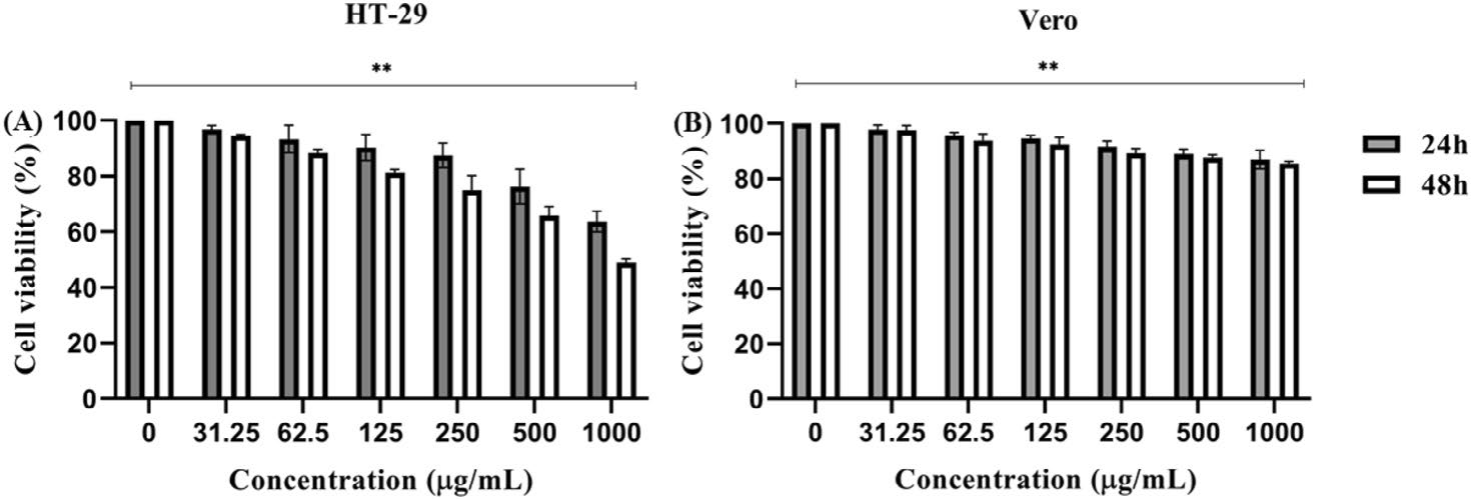
(A) The selective cytotoxic activity of the extract (31.25–10,000 μg/mL) on HT‐29, and (B) Vero cells for 24–48 h. MTT assay was performed to assess cell viability. Data were represented as means ± SD. Statistical differences were analyzed using one‐way ANOVA and Tukey's test for post hoc comparisons. **p < 0.01.

To investigate the selective cytotoxic activity of the *F. pinicola* extract, we treated the HT‐29 CRC cell lines and the Vero kidney cells as a normal cell line. First, the effect of different doses (31.25–1000 μg/mL) of *F. pinicola* extract was examined on the viability of HT‐29 and Vero cells over 24 and 48 h using an MTT assay (Figure 10). As shown in Figure 10, treatment with the extract resulted in a dose‐ and time‐dependent reduction in the viability of HT‐29 cells in varying degrees. However, no effective change was observed in the viability of Vero cells after either 24 or 48 h of treatment with the extract.

The effect of the extract on cell viability was calculated using the dose–response curves generated for each cell line and expressed as an IC_50_ value (Table 6). The SI value is a key parameter in studies investigating the biological activities of substances, especially natural or new compounds. It is defined as the ratio between the toxic concentration of a sample and its effective biologically active concentration. The SI value is a valuable tool for researchers to assess the safety and potential therapeutic benefit of a substance. As a result, FP showed cytotoxic activity in the HT‐29 CRC cell line but did not significantly affect cell viability in the normal epithelial cell line.

**TABLE 6:** IC_50_ (μg/mL) and SI values of the extract.

	HT‐29	Vero	SI^a^
Extract (μg/mL)			
24 h	> 1000	> 2000	2.12
48 h	846 ± 54	> 2000	2.68

^a^ The selectivity index was calculated from IC_50_ values in the normal and cancer cell lines obtained in each independent experiment.

## 4 Discussion

The antimicrobial potential of *F. pinicola* is widely recognized and has demonstrated efficacy against a wide range of microbial pathogens. Previous research has highlighted the remarkable activity of its ethyl acetate and methanol extracts, which at certain concentrations have shown inhibitory activity comparable to standard antibiotics such as gentamycin. These extracts have shown strong activity against Gram‐positive bacteria, including *Bacillus subtilis* and *S. aureus* , as well as various fungal species (Pala et al. 2019). In the present study, the ethanolic extract of *F. pinicola* was tested for its antimicrobial properties, particularly against the *S. aureus* strains ATCC 25923 and ATCC 43300. The extract showed significant activity at a relatively low concentration of 312.5 μg/mL.

Ethanolic extracts obtained from different mycelial cultures of *F. pinicola* have shown variable antimicrobial activity against *S. aureus* . Previous studies, such as Dresch et al. (2015), reported MICs for *F. pinicola* extracts against *S. aureus* ATCC 25923 in the range of 31 to 500 μg/mL, emphasizing the strong antibacterial properties of the extract. In line with these results, the MIC values for the ethanolic extract of *F. pinicola* observed in the current study are exactly in this range, emphasizing its efficacy as an antibacterial agent.

*F. pinicola* contains several bioactive compounds that contribute to its antimicrobial potential. For example, the remarkable effect of triterpenoids on the Gram‐positive species *Bacillus cereus* and *Bacillus subtilis* is noteworthy (Keller et al. 1996; Liu et al. 2010). Triterpenoids are known to interfere with bacterial cell membranes, leading to changes in permeability and bacterial lysis (Raza et al. 2024). As studies on the antimicrobial activities of the triterpenoids of *F. pinicola* are limited, the triterpenoids of the medicinal mushroom support the hypothesis that these compounds interfere with bacterial cell membranes, leading to increased permeability and cell lysis.

Time‐kill assays provide valuable insights into the relationship between antimicrobial concentration and bacterial growth dynamics beyond the information provided by the MIC alone. By revealing the rate and extent of bacterial eradication and distinguishing between bacteriostatic and bactericidal effects, these assays are essential for optimizing dosing strategies and understanding the pharmacodynamics of antimicrobial agents (Foerster et al. 2016). The results of this study show that the ethanolic extract of *F. pinicola* has both bacteriostatic and bactericidal effects against the tested strains of *S. aureus* (ATCC 25923 and ATCC 43300), depending on the concentration used. At the MIC (312.5 μg/mL) and the 2 × MIC, the bacterial growth of both *S. aureus* strains was slowed but not completely suppressed (Table 1, Figure 2). This indicates a bacteriostatic effect in which the extract inhibits bacterial growth without causing immediate cell death. The gradual decrease in bacterial survival suggests that the extract interrupts cellular processes or metabolic activity, preventing proliferation but not killing the cells rapidly. In 4 × MIC, the ethanolic extract showed a rapid bactericidal effect (Table 1, Figure 2). These results suggest that at higher concentrations, the extract transitions from growth inhibition to active killing of bacteria, which is a hallmark of bactericidal activity. The results of the time‐kill assay show a clear concentration‐dependent transition from bacteriostatic to bactericidal activity. This is consistent with the behavior of many natural antimicrobial agents, where lower concentrations impair bacterial growth, while higher concentrations cause irreversible damage to cellular structures or critical processes (Stan et al. 2021; Noumi et al. 2022).

The increasing spread of multidrug‐resistant (MDR) bacteria poses a major challenge to global health and necessitates research into alternative therapeutic strategies. Recent studies have emphasized the urgent need for novel antimicrobial sources, especially from natural products, due to increasing antibiotic resistance (Aldarhami et al. 2023; Almuzaini et al. 2024). Although there is no report in the literature on the efficacy of the natural constituents of *F. pinicola* in combination with antibiotics against multidrug‐resistant bacteria, recent studies have emphasized the potential of mushroom extracts to improve the efficacy of conventional antibiotics through synergistic interactions (Liktor‐Busa et al. 2016; Datta et al. 2020). The MRSA strain ATCC 43300 is a remarkable clinical isolate characterized by its resistance to methicillin and oxacillin. MRSA has gradually developed resistance to several conventional antibiotics, including macrolides, linezolid, ampicillin, streptomycin, sulphonamides, vancomycin, chloramphenicol, and tetracycline, which poses major challenges for the treatment of infections caused by this pathogen (Kali 2015). In this study, all antibiotics in combination with *F. pinicola* extract showed a strong synergism. Considering the oxacillin resistance of *S. aureus* ATCC 43300 (resistance; ≥ 4 μg/mL, MIC: 256 μg/mL), the effective concentration of oxacillin in combination with the extract was found to decrease to 4 μg/mL (Table 2).

Despite the potential antibiofilm properties of *F. pinicola* , there are no studies in the current literature directly linking this macrofungus with antibiofilm activity. However, *F. pinicola* , a well‐known polypore fungus, has been extensively studied for its bioactive compounds, especially its phenolic acids, triterpenoids, and polysaccharides, which have antimicrobial, antioxidant, and therapeutic properties (Cheng et al. 2008; Nowacka et al. 2014). The antibiofilm potential of related fungi is often based on such bioactive secondary metabolites, suggesting that *F. pinicola* may have similar capabilities. For example, phenolic compounds are known to disrupt quorum sensing in bacterial biofilms, while triterpenoids can destabilize biofilm matrices, although specific studies on *F. pinicola* have not yet been documented (Vunduk et al. 2023; Bhattacharya et al. 2023). This study has shown that the ethanolic extract of *F. pinicola* exhibits antibiofilm activity against *S. aureus* strains independent of its antimicrobial activity. This is since the extract significantly inhibited biofilm formation on both polystyrene and stainless‐steel surfaces at concentrations below the MIC, even at concentrations of MIC/32, MIC/64 (Tables 3 and 4, Figures 3 and 5). The antibacterial effect at subinhibitory concentrations is thought to be related to changes in bacterial adherence and membrane integrity rather than direct bactericidal effects. For example, the presence of phenolic compounds in mushroom extracts may alter the expression of genes related to biofilm formation and motility, thereby reducing the ability of bacteria to adhere and form structured communities (Solmaz et al. 2013; Alves et al. 2014). Certain extracts from medicinal mushrooms have shown significant disruptive effects on established biofilms (Solmaz et al. 2013). However, in our study, the ethanolic extract of *F. pinicola* was not able to remove the accumulated biofilm matrix on polystyrene surfaces, even at concentrations above the MIC (2 × MIC, 4 × MIC, Figure 4). Although the extract was not able to disperse the biofilm matrix adhering to the surface, it effectively killed the cells within the already established biofilms. A two‐fold MIC concentration for *S. aureus* ATCC 25923 and a four‐fold MIC concentration for *S. aureus* ATCC 43300 resulted in complete elimination of the cells embedded in the biofilm (Table 5, Figure 6).

*C. violaceum* synthesizes a unique purple pigment known as violacein, which has antimicrobial properties. This pigment not only serves as a distinctive recognition marker for the bacterium, but also plays a crucial role in the study of quorum sensing mechanisms and interactions between microorganisms in different environments (Venkatramanan and Nalini 2024). Bioactive compounds from wild mushrooms, including terpenoids, polysaccharides, and phenolic substances, can disrupt quorum sensing processes that are essential for the stability of biofilms and microbial adhesion (Prajapati et al. 2023; Vunduk et al. 2023; Roychoudhury et al. 2024). Although there is no report in the literature on the mechanisms of the anti‐quorum sensing effect of active compounds from wild mushrooms, and that the active compounds of *F. pinicola* can show an anti‐quorum sensing effect, this study is the first that displays a very strong anti‐quorum sensing effect of the ethanolic extract. Even at sub‐MIC concentrations, an 85.01% decrease in pigment production was observed in strain *C. violaceum* ATCC 12472 (Figure 7).

Efflux pumps are crucial components in the mechanisms of antibiotic resistance of *S. aureus* , especially in its methicillin‐resistant variant (MRSA). These transporter proteins actively excrete a broad spectrum of antimicrobial agents from the bacterial cells, thereby reducing the efficacy of antibiotics and favoring the development of MDR (Zimmermann et al. 2019). Ethidium bromide acts as a substrate for several bacterial efflux pumps and is therefore a valuable tool for evaluating the activity of efflux pumps (Couto et al. 2008). This research suggests that natural mushroom extracts may contain bioactive compounds that act as natural efflux pump inhibitors. However, there are no specific studies directly linking *F. pinicola* to the inhibition of bacterial efflux pumps. Terpenes, which are highly abundant in *F. pinicola* and found in various natural products, including mushrooms, have been identified as effective inhibitors of efflux pumps. In addition, these compounds have the potential to synergize with antibiotics against resistant strains (Liu et al. 2010; Dias et al. 2022). The increase in intracellular accumulation of EtBr observed in the multidrug‐resistant *S. aureus* ATCC 43300 strain exposed to *F. pinicola* extract at sub‐MIC concentrations indicates that the extract disrupts the efflux pump (Figure 8).

The *agrA* and *hla* genes are essential for the virulence and pathogenicity of *S. aureus* , a major pathogen affecting both humans and animals. The *agrA* gene is a key component of the accessory gene regulator (agr) system, a quorum‐sensing mechanism that controls the expression of virulence factors in *S. aureus* (Sully et al. 2014). This previous research has shown that natural compounds, especially those derived from fungi, have an antivirulence effect. Terpenoids and phenolic substances, which are also found in various fungal species, have been shown to interfere with quorum sensing and inhibit biofilm formation in *S. aureus* (Bodede et al. 2018). Similarly, the bioactive metabolites also found in *F. pinicola* , including terpenoids, can interfere with quorum sensing pathways, leading to reduced expression of virulence genes (Bedlovičová and Strapáč 2022). The results of this study indicate that sub‐MIC concentrations of *F. pinicola* extract significantly reduce the expression of key virulence‐associated genes, *agrA* and *hla*, in *S. aureus* . Importantly, this reduction depends on the concentration of the extract and does not affect the viability of the bacteria, as confirmed by CFU (Figure 9).

The present study provides important evidence for the antibacterial, antibiotic promoting, anti‐quorum sensing, and antivirulence properties of *F. pinicola* ; however, the exact molecular pathways controlling these activities remain to be fully elucidated. FT‐IR studies and current research suggest that terpenoids, triterpenoids, and phenolic chemicals in the extract can disrupt bacterial membranes, quorum sensing mechanisms, and virulence gene production. While these data are consistent with the previously documented effects of these classes of compounds, since no purification of the compounds and no molecular docking or omics‐based assays were performed in this study, the postulated mechanisms of action are questionable. Nonetheless, the discovered mechanisms of quorum sensing inhibition, efflux pump disruption, and virulence gene suppression are interrelated processes that together enhance the antimicrobial activity of the extract. Disruption of quorum sensing, indicated by inhibition of violacein and downregulation of *agrA*, can hinder communication between cells, thereby reduce the development of biofilms. Inhibition of efflux pump activity likely increases intracellular accumulation of both natural and synthetic antimicrobials, while downregulation of critical virulence genes such as *hla* further reduces *S. aureus* pathogenicity. Overall, these results demonstrate a multifactorial approach that not only inhibits bacterial multiplication but also impairs critical functions such as biofilm formation and resistance, making *F. pinicola* a viable candidate for combating biofilm‐related and multidrug‐resistant diseases. Subsequent studies using isolated chemicals and transcriptomic or proteomic methods will be crucial to validate the specific biological targets.

While FTIR spectroscopy is proficient at detecting functional groups within a chemical, it does not yield comprehensive structural elucidation. This is because FTIR spectroscopy mainly provides insights into the molecular vibrations and does not reveal the complete arrangement of atoms (Grdadolnik 2002). Nevertheless, the specific IR bands (2938, 1700, 1456, and 1375 cm^−1^, Figure S1) obtained in this study for the ethanolic extract of *F. pinicola* could indicate the triterpenoids and terpenoids identified in *F. pinicola* . The band at 2938 cm^−1^ corresponds to C‐H stretching vibrations, which indicate aliphatic chains that could be characteristic of the triterpenoid structures identified in *F. pinicola* . The band at 1700 cm^−1^ is also indicative of C=O stretching vibrations commonly found in ketones, aldehydes, or carboxylic acids, which could be functional groups in many triterpenoids and sesquiterpenoids. The bands at 1456 and 1375 cm^−1^ are also associated with C‐H bending and C‐O stretching vibrations, respectively, which are typical of alcohols and ethers, further supporting the presence of triterpenoids and terpenoids (Peng et al. 2019; Tai et al. 2019).

Research has shown that ethanolic extracts from *F. pinicola* can induce programmed cell death (apoptosis) in various malignant diseases. In vitro experiments have shown that this extract effectively reduces cell viability in various cancer cell lines, including colon cancer (HCT‐116, HT‐29), breast cancer (MDA‐MB‐231) and lung cancer (A549). The observed cytotoxicity is dose‐dependent, with higher extract concentrations correlating with greater cell death. This anti‐cancer effect appears to involve both the induction of apoptosis and the suppression of tumor cell migration. Remarkably, the extract exhibits selective toxicity and significantly reduces the viability of cancer cells while sparing normal cells, indicating a targeted mode of action (Wang et al. 2014; Wu et al. 2014).

Although the ethanolic extract of *F. pinicola* showed remarkable antibacterial, antibiofilm, anti‐quorum sensing, and antivirulence properties, certain limitations must be taken into account. The work was conducted exclusively in vitro, and although these results provide important preliminary findings, additional in vivo research is required to validate the performance under physiological conditions. Chemical investigation of the extract was limited to FT‐IR spectroscopy, which indicated the presence of triterpenoids and similar substances, but did not allow precise structural identification. Notwithstanding these limitations, the results provide a solid basis for subsequent studies aimed at identifying active elements and testing their therapeutic efficacy using more sophisticated biological models.

## 5 Conclusions

In this study, the ethanolic extract of *F. pinicola* showed significant antimicrobial and selective cytotoxic activities, emphasizing its potential as a therapeutic agent. The extract's ability to inhibit biofilm formation, to disrupt bacterial efflux pumps, and down‐regulate key virulence genes suggests promising applications in the fight against multi‐drug resistant bacteria. Although the results are encouraging, in vivo validation and further toxicity studies are essential to confirm the safety and efficacy of the extract. Future research should focus on the isolation and characterization of its specific bioactive compounds to better understand the mechanisms of action. Overall, this study provides a solid foundation for the potential development of antimicrobial agents from *F. pinicola* .

## Author Contributions

**Başar Karaca:** conceptualization (lead), data curation (equal), funding acquisition (equal), investigation (equal), supervision (lead), writing – original draft (lead). **Noah Kyalo Kilonzo:** data curation (equal), funding acquisition (equal), methodology (equal). **Şilan Korkmaz:** data curation (equal), methodology (equal). **Okan Onar:** data curation (equal), methodology (equal). **Özlem Yıldırım:** supervision (supporting), writing – original draft (supporting). **Arzu Çöleri Cihan:** supervision (supporting), writing – original draft (supporting).

## Ethics Statement

The authors have nothing to report.

## Conflicts of Interest

The authors declare no conflicts of interest.

## Supporting information


**Figure S1.** IR spectrum plot displaying specific bands.

## Data Availability

Data will be made available on request.
