# Supplementary material for: The Dual Role of the Medicinal Mushroom Fomitopsis pinicola in Inhibiting Biofilm and Reducing Antibiotic Resistance of Methicillin‐Resistant Staphylococcus aureus
Source: Food Sci Nutr. 2025 May 27;13(6):e70355. doi: 10.1002/fsn3.70355 (PMC12116333; doi:10.1002/fsn3.70355)
Supplement: Supplementary file 1 — Figure S1. IR spectrum plot displaying specific bands. [file FSN3-13-e70355-s001.docx]

**Supplementary Material**

**Food Science & Nutrition**

**The dual role of the medicinal mushroom *Fomitopsis pinicola* in inhibiting biofilm and reducing antibiotic resistance of methicillin-resistant *Staphylococcus aureus***

**Başar Karaca^a*^, Noah Kyalo Kilonzo^a^, Şilan Korkmaz^a^, Okan Onar^a^, Özlem Yıldırım^a^, Arzu Çöleri Cihan^a^**

**^a^*Department of Biology, Faculty of Science, Ankara University, Ankara 06100, Turkey***

**Corresponding author. Department of Biology, Faculty of Science, Ankara University, Ankara 06100, Turkey.**

**E-mail address:** [**karaca@ankara.edu.tr**](mailto:karaca@ankara.edu.tr) **(Başar Karaca).**


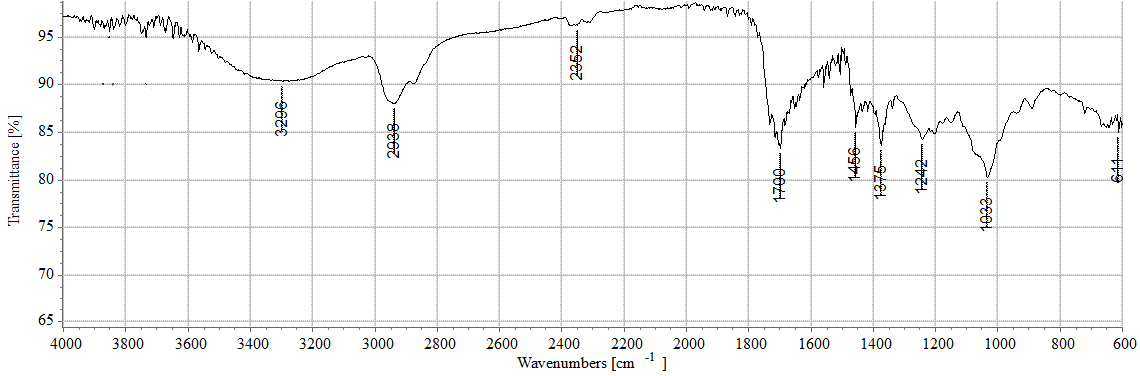


**Fig. S1.** IR spectrum plot displaying specific bands.
